# Molecular Network-Guided Alkaloid Profiling of Aerial Parts of *Papaver nudicaule* L. Using LC-HRMS

**DOI:** 10.3390/molecules25112636

**Published:** 2020-06-05

**Authors:** Kwangho Song, Jae-Hyeon Oh, Min Young Lee, Seok-Geun Lee, In Jin Ha

**Affiliations:** 1 Korean Medicine Clinical Trial Center (K-CTC), Kyung Hee University Korean Medicine Hospital, Seoul 02454, Korea; siwcazb0@gmail.com (K.S.); papermint221@gmail.com (M.Y.L.); 2 Gene Engineering Division, National institute of Agricultural Sciences, Rural Development administration, Jeonju-si, Jeollabuk-do 54874, Korea; jhoh8288@korea.kr; 3 Department of Science in Korean Medicine, KHU-KIST Department of Converging Science & Technology, and Bionanocomposite Research Center, Kyung Hee University, Seoul 02447, Korea; 4 Department of Clinical Korean Medicine, Graduate School, Kyung Hee University, Seoul 02447, Korea

**Keywords:** *Papaver nudicaule*, alkaloids, LC-MS/MS chemical profiling, GNPS molecular networking

## Abstract

*Papaver nudicaule* L. (Iceland poppy) is widely used for ornamental purposes. A previous study demonstrated the alleviation of lipopolysaccharide-induced inflammation mediated by *P. nudicaule* extract through nuclear factor-kappa B and signal transducer and activator of transcription 3 inactivation. As isoquinoline alkaloids are chemical markers and bioactive constituents of *Papaver* species, the present study investigated the alkaloid profile of aerial parts of five *P. nudicaule* cultivars with different flower colors and a *P. rhoeas* cropped for two years. A combination of liquid chromatography high-resolution mass spectrometry and molecular networking was used to cluster isoquinoline alkaloids in the species and highlight the possible metabolites. Aside from the 12 compounds, including rotundine, muramine, and allocryptopine, identified from Global Natural Products Social library and reported information, 46 structurally related metabolites were quantitatively investigated. Forty-two and 16 compounds were proposed for chemical profiles of *P. nudicaule* and *P. rhoeas*, respectively. Some species-specific metabolites showed similar fragmentation patterns. The alkaloid abundance of *P. nudicaule* differed depending on the flower color, and the possible chemical markers were proposed. These results show that molecular networking-guided dereplication allows investigation of unidentified metabolites. The derived chemical profile may facilitate evaluation of *P. nudicaule* quality for pharmacological applications.

## 1. Introduction

Plants in the *Papaver* L. genus from *Papaveraceae* (commonly known as poppy) family have been used for traditional medicinal practices and ornamental purposes for a long time. *Papaver rhoeas* L. (Corn poppy) and *Papaver nudicaule* L. (Iceland poppy) are widely used for ornamentation owing to their multicolored flowers. Nudicaulins, which are flavonoid-derived indole alkaloids, along with pelargonidin, kaempferol, and gossypetin glycosides, are responsible for the white, red, orange, and yellow colored petals of different *P. nudicaule cultivars* [1,2]. *P. nudicaule* belongs to the Papaveraceae family, and is an annual herbaceous species. This plant can produce various alkaloids, particularly isoquinoline alkaloids (IAs) [3]. IAs from *Papaver* species possess potent pharmacological properties, including narcotic analgesic, antimicrobial, muscle relaxant, cough suppressant, and anticancer effects [4,5]. However, studies on chemical constituents and biological activities of *Papaver* species have mainly been performed on *P. somniferum,* which produces morphine alkaloids [6].

To understand the metabolites of other *Papaver* species and their biological activities, the metabolite profiling of IAs in *P. rhoeas* was previously performed using liquid chromatography (LC) coupled with quadrupole time-of-flight (Q-TOF) mass spectrometry (MS) [7]. *P. nudicaule* extracts have anti-inflammatory activity and decrease the inflammatory response induced by lipopolysaccharide by inhibiting the nuclear factor-kappa B (NF-κB) and signal transducer and activator of transcription 3 (STAT3) signaling pathway in the previous report [8]. The study proposed that IAs, such as allocryptopine, from *P. nudicaule* could be the active constituents and a representative subset for this species. However, IAs of *P. nudicaule* have been rarely reported, except for flavonols; indole alkaloid skeleton of yellow petals [9,10,11] and some alkaloids of aerial parts, including 8, 14-dihydroflavinantine, pseudoprotopine, allocryptopine, dihydroamuronine, amuronine, amurensinine *N*-oxide A, and (−)-amurensinine *N*-oxide B [12,13].

Alkaloid profiling of *P. nudicaule* is crucial to investigate its pharmaceutical potential. However, a major challenge for MS-based chemical profiling of *P. nudicaule* is the identification of alkaloids. To the best of our knowledge, the compound information and MS/MS spectra of *P. nudicaule* have been rarely reported. Our main interest is to highlight the species-specific metabolites and propose a reliable alkaloid profile. Therefore, this study utilized the Global Natural Products Social (GNPS) molecular networking platform and significantly accelerated the MS/MS-based clustering of alkaloids, even for compounds with no spectral matches in any public database or in-house MS/MS library [14,15]. 

This study aimed to identify IAs and investigate their abundance between *P. nudicaule* and *P. rhoeas*, and further analyze the chemical markers in specimens from aerial parts of five cultivars of *P. nudicaule* based on liquid chromatography high-resolution mass spectrometry (LC-HRMS) and molecular network analysis.

## 2. Results

### 2.1. Molecular Network-Based Annotation of Species-Specific Metabolites

**Figure 1:**
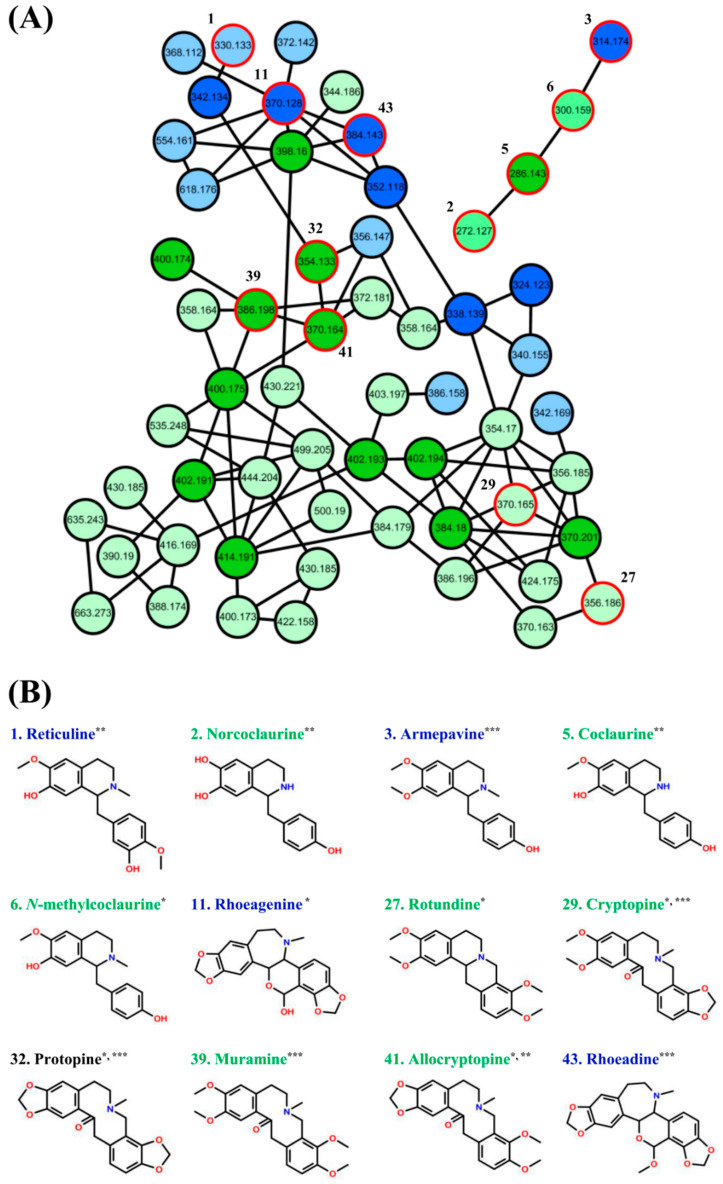
(**A**) Alkaloid-targeted molecular network generated from the ethanolic extracts of *P. nudicaule* and *P. rhoeas*. Colors inside nodes represent species-specific metabolites: green, *P. nudicaule*; blue, *P. rhoeas*. (**B**) Nodes with red outer circles represent ions dereplicated by GNP libraries (*), authentic compounds (**), and literature (***). Identified ions are presented with their common names and chemical structures.

To organize the compound candidates for the alkaloid profiling of *P. nudicaule* and *P. rhoeas*, the LC-MS/MS data from 12 extracts was submitted to molecular networking through the GNPS web platform (http://gnps.ucsd.edu, ID: d38cc1e769524002ad2c2122f7375f08). The 12 extracts were prepared from the five cultivars of *P. nudicaule* with different flower colors (orange, purple, scarlet, white, and yellow), and a *P. rhoeas* sample was collected for two years (2017 and 2018). The extract from aerial parts of *P. nudicaule* with white bloom in 2017 is abbreviated as 17NW. The same format is followed for other extracts (i.e., orange, NO; purple, NP; scarlet, NS; and yellow, NY). The extract of aerial parts of *P. rhoeas* collected in 2018 is abbreviated as 18RA. The resulting molecular network comprised 2121 molecular features and 102 independent clusters with at least three features (Supplementary Figure S1). A total of six alkaloids were identified through MS/MS library matching of GNPS. The nodes belonged to two molecular clusters (Figure 1A). The two clusters comprising 58 molecular features were selected as species-specific alkaloids from the chemical profiles of *P. nudicaule* and *P. rhoeas*. This strategy was based on molecular networking using spectral similarity that allowed grouping of structurally related metabolites [16]. Although the molecular networking platform significantly increased structural annotations and clustering of *Papaver* species, the MS/MS library matching unfortunately failed to cover all metabolites. Indeed, 44 IAs were identified from *P. rhoeas* in the previous report [7], but the molecular network-guided profiling in this study missed some of these alkaloids. The unidentified alkaloids belong to other clusters that are not yet characterized are speculated.

Within the 58 metabolites, six alkaloids were further annotated by manually comparing their MS/MS spectra with the in-house MS/MS library and previous literature [17,18]. The molecular network-guided approach reduced this laborious step by narrowing down the possible MS/MS features. Twelve alkaloids were identified (Figure 1B), and 46 metabolites were proposed to be structurally related alkaloids. Table 1 summarizes the chromatographic information (retention times), high-resolution MS/MS-based information (observed mass, formulas, MS/MS fragment ions), and quantitative information (abundant species, fold changes in peak areas) of each metabolite.

### 2.2. Characterized Alkaloids of P. nudicaule

**Figure 2:**
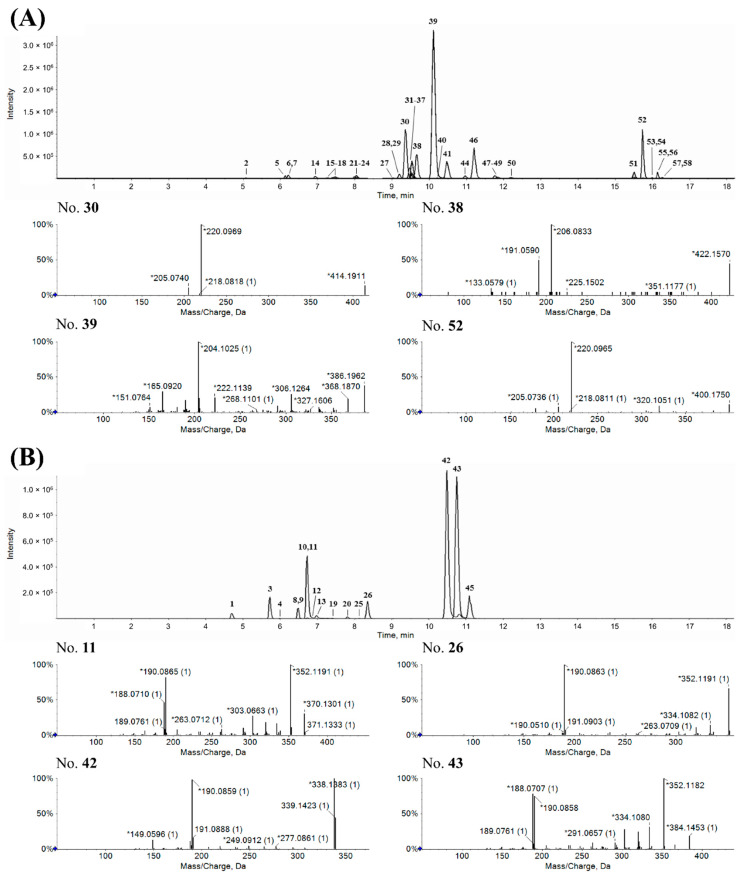
Representative extracted ion chromatograms of *P. nudicaule* (**A**) and *P. rhoeas* (**B**) based on clustered alkaloids. Specific metabolites of each species were numbered according to their retention times, and the fragmentation patterns of major peaks are presented.

Representative chromatograms for the selected IAs are shown in Figure 2. The major metabolites of *P. nudicaule* revealed some specific molecular ions upon MS/MS fragmentation. As shown in Figure 2A, compounds **30** and **52** generated fragment ions at *m/z* 220.10, whereas compounds **38** and **39** generated fragment ions at *m/z* 206.08 and *m/z* 204.10, respectively. The molecular ions at *m/z* 204.10, *m/z* 206.08, *m/z* 206.12 (data not shown), and *m/z* 220.10 could be diagnostic ions of *P. nudicaule*, as most major metabolites generated one of the fragment ions (Table 1). However, the major metabolites of *P. rhoeas* (compounds **11**, **26**, **42**, and **43**) were mainly fragmented to molecular ions with *m/z* 190.09 (Figure 2B).

**Figure 3:**
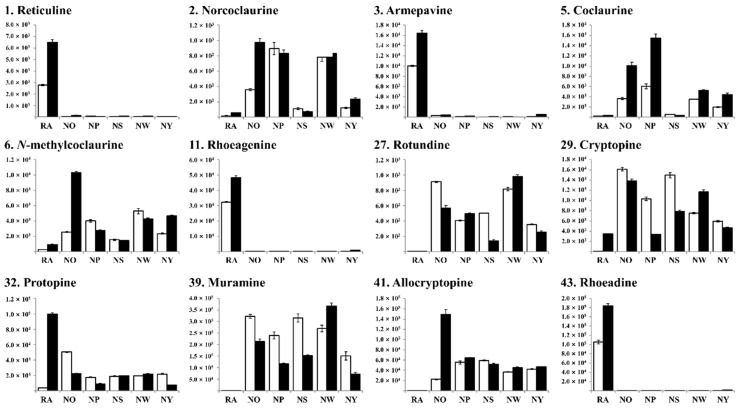
The abundance of the identified alkaloids in *P. nudicaule* and *P. rhoeas*, as determined by liquid chromatography high-resolution mass spectrometry (LC-HRMS). Bars with white and black colors represent specimens of 2017 and 2018, respectively. Reticuline (**1**), demethylcoclaurine (**2**), armepavine (**3**), coclaurine (**5**), *N*-methylcoclaurine (**6**), rhoeagenine (**11**), rotundine (**27**), cryptopine (**29**), protopine (**32**), muramine (**39**), allocryptopine (**41**), and rhoeadine (**43**).

A relative quantification analysis was performed to explore the alkaloid abundance in *P. nudicaule* and *P. rhoeas*. Among the 58 compounds within two molecular clusters, 42 and 16 were abundant in *P. nudicaule* and *P. rhoeas*, respectively. As shown in Supplementary Table S1, the peak areas of each compound and the relative fold change between the two species were calculated. Considering the mean peak areas of *P. nudicaule* samples, species-specific alkaloids are summarized in Table 1 depending on fold change values. Furthermore, 12 identified alkaloids were quantitatively compared based on their peak areas. Figure 3 presents their abundance in 12 specimens harvested for two years (2017 and 2018) comprising five cultivars of *P. nudicaule* blooms and a *P. rhoeas*. Reticuline (**1**), armepavine (**3**), rhoeagenine (**11**), and rhoeadine (**43**) were more abundant in *P. rhoeas*. The levels of demethylcoclaurine (**2**), coclaurine (**5**), *N*-methylcoclaurine (**6**), rotundine (**27**), muramine (**39**), and allocryptopine (**41**) were much higher in *P. nudicaule* (Figure 3). Protopine (**32**) was detected in both species among protopine-type alkaloids.

### 2.3. Alkaloid-Based Multivariate Analysis

**Figure 4:**
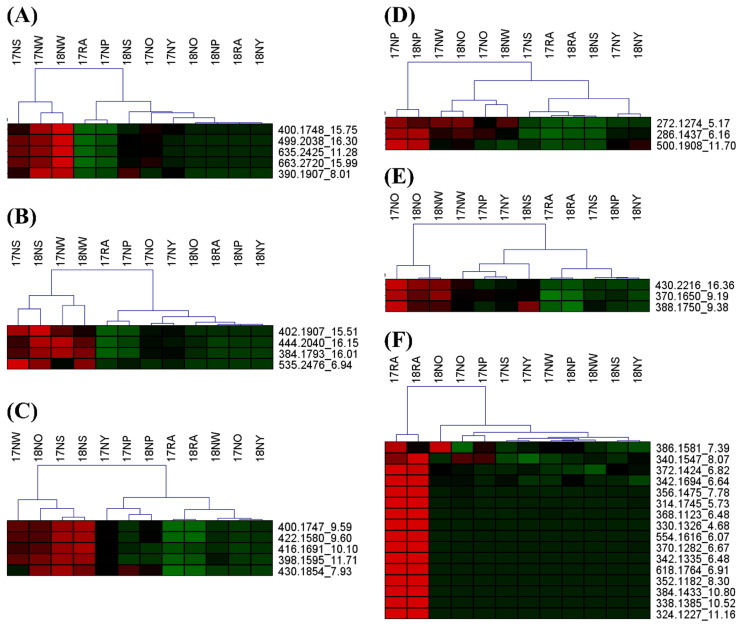
Heatmaps of the molecular features illustrating K-means clustering for the 58 alkaloids of *P. nudicaule* and *P. rhoeas* derived from molecular network-guided analysis. Specimens from each analysis were hierarchically clustered. Representative metabolites were proposed for (**A**) NW, (**B**) NS and NW, (**C**) NS, (**D**) NP, (**E**) NO, and (**F**) RA.

A multivariate analysis was performed for 12 specimens based on molecular network-guided IAs. Figure 4 presents the K-means clustering for 58 identified alkaloids. Representative compounds are proposed. Compounds **23**, **46**, **52**, **53**, and **57** were significantly abundant in NW (Figure 4A), while compounds **14**, **51**, **54**, and **55** were abundant in both NW and NS (Figure 4B). Compounds **21**, **37**, **38**, **40**, and **48** in NS (Figure 4C); **2**, **5**, and **47** in NP (Figure 4D); and **29**, **31**, and **58** in NO (Figure 4E) could be representative metabolites. None was represented for the specimen with yellow colored flower. Sixteen metabolites were significantly abundant in *P. rhoeas* as compared with those in *P. nudicaule* (Figure 4F), suggesting that alkaloid production in the two species depends on different biosynthesis pathways and that, even within the same species, the abundance of IAs is very different for each *P. nudicaule* cultivar depending on flower color. These results further the understanding of the biosynthesis of alkaloids in *P. nudicaule*, and the quantitative variation may explain the difference in biological activities within the same species. Protopine detected in both *P. rhoeas* and *P. nudicaule* (Figure 3) is also one of the IAs found in *Sanguinaria canadensis* L., another species in the family Papaveraceae. Protopine has various pharmacological and biological activities, including anti-inflammatory, anti-infectious, neuroprotective, and antithrombotic effects [19]. Further investigations should address the correlation between their chemical composition and biological activities.

## 3. Materials and Methods

### 3.1. Plant Material 

The aerial parts of *P. nudicaule* L. and *P. rhoeas* L. were harvested by the Korea National Academy of Agricultural Science, Rural Development Administration. The five cultivars of *P. nudicaule* with different flower colors (orange, purple, scarlet, white, and yellow) and *P. rhoeas* samples collected for two years (2017 and 2018) were immediately frozen in liquid nitrogen and stored at −70°C in a deep freezer. For each specimen, experiments were performed in triplicate under the same conditions. 

### 3.2. Reagents and Chemicals

The suppliers of the reference standard for IAs were previously described [5]. High-performance liquid chromatography (HPLC)-grade acetonitrile and methanol were purchased from Honeywell Burdick and Jackson (Morristown, NJ, USA). Formic acid and ammonium formate were procured from Sigma-Aldrich (St. Louis, MO, USA). Deionized water was obtained using a pure water purification system (Human Co., Seoul, Korea).

### 3.3. Sample Preparation

Aerial parts of lyophilized *Papaver* species were ground into a fine powder. Two grams of all samples were ultrasonicated in 5 mL of ethanol for 30 min. The supernatants were filtered through 0.2 μm polytetrafluoroethylene syringe filters and dried using a SAVANT SPD2010 SpeedVac^TM^ concentrator (Thermo Scientific, Asheville, NC, USA). Ethanolic extracts were dissolved in HPLC-grade methanol to 2.0 mg/mL and either directly analyzed or stored at −70°C until analysis.

### 3.4. LC and MS Analysis

The LC-MS system comprised a Vanquish UHPLC system (Thermo Fisher Scientific, Sunnyvale, CA, USA) with an Acquity UPLC HSS T3 column (2.1 mm × 100 mm, 1.7 μm; Waters) and a Triple TOF 5600^+^ mass spectrometer system (Sciex, Foster City, CA, USA). The ultrahigh performance liquid chromatography (UHPLC) system used 0.05% formic acid and 2.5 mM ammonium formate in water as eluent A and acetonitrile as eluent B. The optimized elution program was as follows: 0–2.5 min (1% B), 2.5–3.0 min (1–10% B), 3.0–6.0 min (10–19% B), 6.0–9.0 min (19–22% B), 9.0–14.0 min (22–25% B), 14.0–17.0 min (25–70% B), 17.0–19.0 min (70–100% B), 19.0–22.0 min (100% B), and equilibration with 1% B for 3 min at a flow rate of 0.4 mL/min. The column was maintained at 40 °C, and the auto-sampler was held at 4 °C. The injection volume of each sample solution was 1 μL. The MS/MS data were acquired by an information-dependent acquisition scan at positive-ion mode, and the parameters were as follows: mass range 50–1500 *m/z*, ion spray voltage, 4.5 kV; source temperature, 450 °C; declustering potential, 50 V; nitrogen as nebulizer gas, 50 L/min; heater gas, 50 L/min; curtain gas, 25 L/min; and collision energy, 10 eV.

### 3.5. LC-MS/MS Data Processing

The acquired AB Sciex dataset (.wiff) was directly imported into MZmine 2.53 [20]. Each detection of ms1 and ms2 levels was filtered with ions showing a minimum height 1.0 × 10^3^ and 5, respectively, and the extracted ion chromatograms (XICs) were built with ions showing a minimum time span of 0.02 min, a minimum height of 1.0 × 10^3^, and an *m/z* tolerance of 0.002 Da (or 10.0 ppm). The chromatograms were deconvoluted by a baseline cut-off algorithm using the following parameters: minimum peak height 1.0 × 10^3^, peak duration range 0.02–0.25 min, and baseline level 1.0 × 10. The deconvoluted peaks were deisotoped using an isotopic peaks grouper algorithm with an *m/z* tolerance of 0.002 Da (or 10.0 ppm) and a retention time tolerance of 0.1 min, and aligned together into a peak list using a join aligner module and following parameters: *m/z* tolerance at 0.005 Da (or 20.0 ppm), weight for *m/z* of 70, absolute retention time tolerance of 0.1 min, and weight for *m/z* of 30. The chromatogram was gap-filled by a peak finder module with an intensity tolerance of 10.0%, *m/z* tolerance of 0.002 Da (or 10.0 ppm), and absolute retention time tolerance of 0.2 min. The processed peak list was eventually exported to an mgf file using the GNPS-FBMN module for GNPS molecular networking [21].

### 3.6. Identification of Species-Specific Metabolites Using Molecular Network Processing

The MZmine-processed LC-MS/MS peak lists were window-filtered by choosing only the top six peaks in the ± 50 Da window throughout the spectrum. A network was created where edges were filtered to have a cosine score > 0.70 and more than four matched peaks. Further edges between two nodes were retained in the network only if each node appeared in every other’s respective top-10 most similar nodes. The spectra in the network were then searched against the spectral library of GNPS. The library spectra were filtered in the same manner as the input data. The molecular network was visualized using Cytoscape 3.7.2 [22]. 

### 3.7. Multivariate Analysis

Identified alkaloids and peak areas were scaled by unit variance based on the year of specimen collection. The scaled data were subjected to K-means clustering and hierarchical tree construction using Multi-experiment Viewer (MeV) version 4.9.0 [23]. The K-means clustering was performed using Pearson’s correlation with 12 cluster numbers and optimized order of molecular features, and the hierarchical trees were constructed using complete linkage clustering with optimized order of sample leaf.

## 4. Conclusions

In the present study, a combination of LC-HRMS and molecular networking was applied to cluster structurally related compounds in natural products and highlight the possible metabolites important for the chemical profiling of the species. As a case study, IAs from *P. nudicaule* and *P. rhoeas* were investigated. The chemical annotation was significantly increased using the molecular networking platform. A total of 58 metabolites were selected and quantitatively analyzed. Forty-two and 16 compounds were proposed as chemical profiles of *P. nudicaule* and *P. rhoeas*, respectively. The alkaloid abundance of *P. nudicaule* was different depending on the color of flowers. These results suggest that the LC-HRMS and molecular networking-guided dereplication method can be a powerful tool for the chemical investigation of unidentified metabolites and chemotaxonomic approaches in the phytomedicine field. Together, these results may contribute to the understanding of the biosynthesis of alkaloids in *P. nudicaule* and the evaluation of the quality of this plant for further pharmaceutical applications.

## Figures and Tables

**Table 1:** Molecular network-based alkaloids of *P. nudicaule* and *P. rhoeas* by liquid chromatography (LC)- quadrupole time-of-flight (QTOF)-mass spectrometry (MS)/MS.

Peak No.	Rt (min)	Observed Mass (Da)	Calculated Mass (Da)	Formula	Adduct Form	MS/MS Fragment Ions (*m/z*)	Species	Fold Change *
1	4.68	330.1326	330.1342	C_1__9_H_2__4_NO_4_	[M + H]+	263.0700, 194.0804 *, 189.0688, 177.0769 *, 176.0703 *	*P. rhoeas*	−1.92
2	5.17	272.1274	272.1287	C_16_H_18_NO_3_	[M + H]+	256.1035 *, 238.0969, 162.0625, 108.0529 *, 107.0501 *	*P. nudicaule*	0.41
3	5.73	314.1745	314.1756	C_19_H_24_NO_3_	[M + H]+	271.1322, 269.1156, 237.0897, 175.0758, 107.0499 *	*P. rhoeas*	−1.84
4	6.07	554.1616	554.1630	C_38_H_22_N_2_O_3_	[M + H]+	352.1170 *, 334.1059, 190.0855 *	*P. rhoeas*	−1.98
5	6.16	286.1437	286.1443	C_17_H_20_NO_3_	[M + H]+	270.1225 *, 238.0931, 210.0994, 108.0535 *, 107.0498 *	*P. nudicaule*	1.10
6	6.27	300.1589	300.1600	C_18_H_22_NO_3_	[M + H]+	270.1193 *, 238.0928, 176.0773, 108.0514 *, 107.0482 *	*P. nudicaule*	1.07
7	6.31	358.1643	358.1655	C_20_H_24_NO_5_	[M + H]+	277.0835, 191.0929, 190.0851 *, 151.0750	*P. nudicaule*	2.28
8	6.48	342.1335	342.1342	C_19_H_20_NO_5_	[M + H]+	263.0708, 235.0753, 206.0803, 176.0704 *, 165.0543	*P. rhoeas*	−1.92
9	6.48	368.1123	368.1134	C_20_H_18_NO_6_	[M + H]+	350.1043, 332.0949, 261.0567, 188.0705 *	*P. rhoeas*	−1.67
10	6.64	342.1694	342.1705	C_20_H_24_NO_4_	[M + H]+	297.1128 *, 282.0899, 265.0851 *, 237.0906, 191.0858	*P. rhoeas*	−0.77
11	6.67	370.1282	370.1291	C_20_H_20_NO_6_	[M + H]+	352.1191 *, 334.1087, 320.0927, 190.0865 *, 188.0710 *	*P. rhoeas*	−3.26
12	6.82	372.1424	372.1447	C_20_H_22_NO_6_	[M + H]+	354.1340, 322.1152, 204.1051 *, 192.1013	*P. rhoeas*	−0.81
13	6.91	618.1764	618.1791	C_39_H_26_N_2_O_6_	[M + H]+	370.1305, 352.1171 *, 321.0745, 190.0835	*P. rhoeas*	−2.60
14	6.94	535.2476	535.2444	C_30_H_35_N_2_O_7_	[M + H]+	518.2158, 504.2009 *, 320.1053, 220.0970 *, 205.0732	*P. nudicaule*	3.35
15	7.11	344.1864	344.1862	C_20_H_26_NO_4_	[M + H]+	298.1471, 267.1026, 191.0932, 190.0876 *	*P. nudicaule*	0.56
16	7.12	358.1641	358.1655	C_20_H_24_NO_5_	[M + H]+	340.1564, 278.0934, 194.0830, 176.0704 *	*P. nudicaule*	0.33
17	7.27	370.1635	370.1655	C_21_H_24_NO_5_	[M + H]+	338.1369, 238.0629, 192.1021 *	*P. nudicaule*	2.07
18	7.38	372.1808	372.1811	C_21_H_26_NO_5_	[M + H]+	354.1692, 291.1015, 222.1118, 204.1017 *, 190.0853	*P. nudicaule*	2.20
19	7.39	386.1581	386.1604	C_21_H_24_NO_6_	[M + H]+	306.1193, 206.1168, 191., 190.0865 *	*P. rhoeas*	−1.67
20	7.78	356.1475	356.1498	C_20_H_22_NO_5_	[M + H]+	340.1547 *, 325.1345, 267.0990, 192.1015 *, 177.0782	*P. rhoeas*	−1.26
21	7.93	430.1854	430.1866	C_23_H_28_NO_7_	[M + H]+	412.1746 *, 350.1138, 220.0961 *, 218.0798, 205.0839	*P. nudicaule*	2.25
22	8.00	402.1930	402.1917	C_22_H_28_NO_6_	[M + H]+	384.1809 *, 335.1278, 206.1175 *, 193.0857, 179.0701	*P. nudicaule*	3.25
23	8.01	390.1907	390.1917	C_21_H_28_NO_6_	[M + H]+	372.1808 *, 310.1199, 208.0969 *, 193.0724	*P. nudicaule*	2.85
24	8.05	356.1859	356.1862	C_21_H_26_NO_4_	[M + H]+	206.1178 *, 190.0868, 162.0911	*P. nudicaule*	2.92
25	8.07	340.1547	340.1549	C_20_H_22_NO_4_	[M + H]+	323.1095, 277.0838, 192.1016 *, 177.0790	*P. rhoeas*	−1.67
26	8.30	352.1182	352.1185	C_20_H_18_NO_5_	[M + H]+	334.1082, 320.0924, 190.0863 *	*P. rhoeas*	−3.99
27	8.97	356.1857	356.1862	C_21_H_26_NO_4_	[M + H]+	325.1384, 249.1838, 192.1017 *, 177.0777	*P. nudicaule*	2.78
28	9.13	400.1745	400.1760	C_22_H_26_NO_6_	[M + H]+	382.1670, 341.1352, 282.1280, 204.1006 *, 165.0901 *	*P. nudicaule*	2.08
29	9.19	370.1650	370.1655	C_21_H_24_NO_5_	[M + H]+	352.1534, 291.1014, 222.1122, 205.1094 *, 204.1018 *	*P. nudicaule*	2.10
30	9.30	414.1901	414.1917	C_23_H_28_NO_6_	[M + H]+	220.0969 *, 205.0740	*P. nudicaule*	3.84
31	9.38	388.1750	388.1760	C_21_H_26_NO_6_	[M + H]+	370.1647 *, 352.1541 *, 336.1232, 322.1189, 308.1273	*P. nudicaule*	2.59
32	9.47	354.1338	354.1342	C_20_H_20_NO_5_	[M + H]+	275.0710, 247.0758, 206.0812, 189.0779 *, 188.0708 *	*P. nudicaule*	0.82
33	9.47	403.1974	403.1995	C_22_H_29_NO_6_	[M + H]+	385.1845, 354.1717, 280.1043, 207.1207 *, 206.1171 *	*P. nudicaule*	3.67
34	9.48	384.1797	384.1811	C_22_H_26_NO_5_	[M + H]+	352.1535, 325.1429, 206.1183 *, 190.0870	*P. nudicaule*	3.83
35	9.48	402.1935	402.1917	C_22_H_28_NO_6_	[M + H]+	384.1807 *, 353.1394, 325.1411, 206.1183 *, 190.0869	*P. nudicaule*	3.73
36	9.49	424.1749	424.1760	C_24_H_26_NO_6_	[M + H]+	384.1911, 214.0871, 206.1176 *	*P. nudicaule*	2.78
37	9.59	400.1747	400.1760	C_22_H_26_NO_6_	[M + H]+	382.1643, 206.0815 *, 191.0586	*P. nudicaule*	3.57
38	9.60	422.1580	422.1604	C_24_H_24_NO_6_	[M + H]+	351.1177, 206.0833 *, 191.0590 *	*P. nudicaule*	2.14
39	10.05	386.1980	386.1968	C_22_H_28_NO_5_	[M + H]+	368.1870, 306.1264 *, 222.1139, 204.1025 *, 190.0872	*P. nudicaule*	3.65
40	10.10	416.1691	416.1709	C_22_H_26_NO_7_	[M + H]+	398.1606, 222.0767 *, 205.0733	*P. nudicaule*	2.41
41	10.48	370.1643	370.1655	C_21_H_24_NO_5_	[M + H]+	290.0944 *, 206.0813, 188.0709 *, 181.0861	*P. nudicaule*	2.72
42	10.52	338.1385	338.1392	C_20_H_20_NO_4_	[M + H]+	277.0861, 249.0912, 190.0859 *, 149.0596	*P. rhoeas*	−1.67
43	10.80	384.1433	384.1447	C_21_H_22_NO_6_	[M + H]+	352.1182 *, 334.1080, 320.0922, 190.0858 *, 188.0707 *	*P. rhoeas*	−4.37
44	11.04	370.2009	370.2018	C_22_H_28_NO_4_	[M + H]+	206.1178 *, 190.0868	*P. nudicaule*	4.08
45	11.16	324.1227	324.1236	C_19_H_18_NO_4_	[M + H]+	250.0942, 176.0707 *, 149.0596 *	*P. rhoeas*	−3.29
46	11.28	635.2425	635.2393	C_37_H_35_N_2_O_8_	[M + H]+	499.2079, 398.1601 *, 380.1486, 220.0967 *	*P. nudicaule*	2.05
47	11.70	500.1908	500.1921	C_26_H_30_NO_9_	[M + H]+	456.2026, 397.1888, 220.0972 *, 205.0742	*P. nudicaule*	0.97
48	11.71	398.1595	398.1604	C_22_H_24_NO_6_	[M + H]+	382.1282 *, 364.1176, 336.1231 *, 193.0859	*P. nudicaule*	3.51
49	11.78	386.1957	386.1968	C_22_H_28_NO_5_	[M + H]+	222.1129 *, 161.0831	*P. nudicaule*	3.33
50	12.15	354.1695	354.1705	C_21_H_24_NO_4_	[M + H]+	338.1361, 190.0864 *, 149.0593	*P. nudicaule*	3.11
51	15.51	402.1907	402.1917	C_22_H_28_NO_6_	[M + H]+	384.1812 *, 322.1200, 220.0975 *, 205.0734	*P. nudicaule*	3.85
52	15.75	400.1748	400.1760	C_22_H_26_NO_6_	[M + H]+	382.1649, 320.1051, 220.0965 *, 205.0736	*P. nudicaule*	3.47
53	16.00	663.2720	663.2706	C_39_H_39_N_2_O_8_	[M + H]+	499.2075, 398.1603 *, 380.1482, 220.0972 *	*P. nudicaule*	2.55
54	16.01	384.1793	384.1811	C_22_H_26_NO_5_	[M + H]+	322.1197, 220.0970 *, 205.0729	*P. nudicaule*	2.77
55	16.15	444.2040	444.2022	C_24_H_30_NO_7_	[M + H]+	384.1814 *, 322.1204, 220.0974 *, 205.0730	*P. nudicaule*	3.51
56	16.18	430.1852	430.1866	C_23_H_28_NO_7_	[M + H]+	370.1661 *, 322.1185, 206.0810 *, 191.0576	*P. nudicaule*	2.35
57	16.30	499.2038	499.2022	C_33_H_27_N_2_O_3_	[M + H]+	351.1147, 320.1046, 220.0969 *, 205.0733	*P. nudicaule*	2.94
58	16.36	430.2216	430.2230	C_24_H_32_NO_6_	[M + H]+	384.1808 *, 353.1377, 206.1173 *, 192.1008	*P. nudicaule*	2.21

* Relative fold changes between the two species were calculated from the ratio of the mean peak areas of *P. nudicaule* samples to that of *P. rhoeas* samples.

## Supplementary Materials

The following are available online, Figure S1: Molecular network from ethanolic extracts of *Papaver nudicaule* and *Papaver rhoeas*; Table S1: LC-MS/MS information for clustered metabolites.
