# Erratum: Song, K.; et al. Molecular Network-Guided Alkaloid Profiling of Aerial Parts of *Papaver nudicaule* L. Using LC-HRMS. *Molecules* 2020, *25*, 2636

**DOI:** 10.3390/molecules26020339

**Published:** 2021-01-11

**Authors:** Kwangho Song, Jae-Hyeon Oh, Min Young Lee, Seok-Geun Lee, In Jin Ha

**Affiliations:** 1 Korean Medicine Clinical Trial Center (K-CTC), Kyung Hee University Korean Medicine Hospital, Seoul 02454, Korea; siwcazb0@gmail.com (K.S.); papermint221@gmail.com (M.Y.L.); 2 Gene Engineering Division, National Institute of Agricultural Sciences, Rural Development Administration, Jeonju-si, Jeollabuk-do 54874, Korea; jhoh8288@korea.kr; 3 Department of Science in Korean Medicine, KHU-KIST Department of Converging Science & Technology, and Bionanocomposite Research Center, Kyung Hee University, Seoul 02447, Korea; 4 Department of Clinical Korean Medicine, Graduate School, Kyung Hee University, Seoul 02447, Korea

The authors wish to make the following change to their paper [1]. The Funding section is incorrect in the published paper. The correct Funding should be as follows: 

This research was supported by a grant from the Basic Science Research Program through the National Research Foundation of Korea funded by the Ministry of Education (NRF-2018R1D1A1B07047610) and was carried out with the support of the “Cooperative Research Program for Agriculture Science and Technology Development (Project No. PJ01184702)” Rural Development Administration, Republic of Korea. 

We apologize for any inconvenience caused to the readers by this mistake.
