# Supplementary material for: Molecular Network-Guided Alkaloid Profiling of Aerial Parts of Papaver nudicaule L. Using LC-HRMS
Source: Molecules. 2020 Jun 5;25(11):2636. doi: 10.3390/molecules25112636 (PMC7321159; doi:10.3390/molecules25112636)
Supplement: Supplementary file 1 [file molecules-25-02636-s001.zip › Supplementary Figure S1.docx]

**Molecular network-guided alkaloid profiling in aerial parts of *Papaver nudicaule* using LC-HRMS**

Kwangho Song^1†^, Jae-Hyeon Oh^2†^, Min Young Lee^1^, Seok-Geun Lee^1,3^*, In Jin Ha^1,4^*

***^1^*** *Korean Medicine Clinical Trial Center (K-CTC), Kyung Hee University Korean Medicine Hospital, Seoul 02454, Republic of Korea*

*^2^* *Genomics Division, Department of Agricultural Biotechnology, National Institute of Agricultural Science (NAS), Rural Development Administration (RDA), Jeollabuk-do, Republic of Korea*

^3^ *Department of Science in Korean Medicine, KHU-KIST Department of Converging Science & Technology, and Bionanocomposite Research Center, Kyung Hee University, Seoul 02447, Republic of Korea*

*^4^* *College of Korean Medicine, Kyung Hee University, Seoul 02447, South Korea*

Prepared for *Molecules*, May 2020

*Corresponding author

^*^E-mail address: seokgeun@khu.ac.kr; ijha0@naver.com

Tel: +82-2-10-2763-7744; Fax: +82-2-958-9597

**Supplementary Figure S1.** Molecular network from ethanolic extracts of *Papaver nudicaule* and *Papaver rhoeas* (GNPS ID: d38cc1e769524002ad2c2122f7375f08). The networks consist of 2121 molecular features. Among 102 independent clusters containing at least 3 features, two clusters (component index 9 and 23) were filtered as alkaloid subclasses by GNP library matching.

**
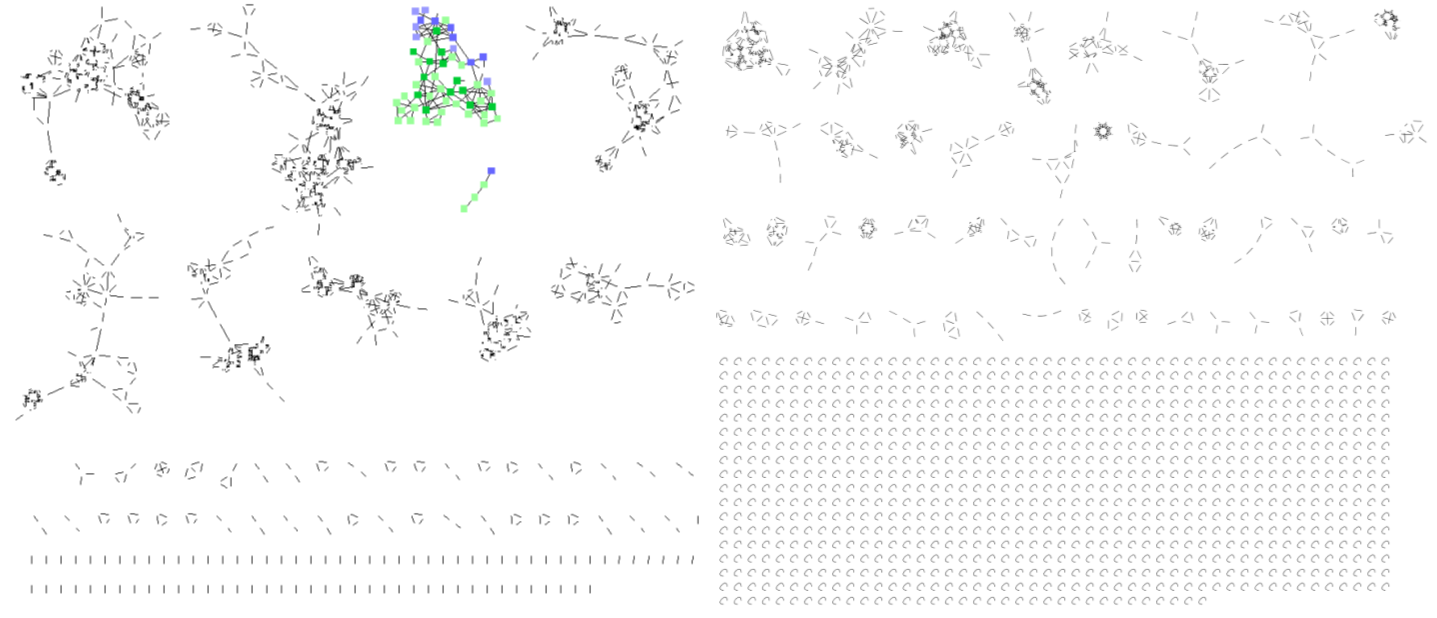
**
